# Evolution or revolution? Changing the way science is published and communicated

**DOI:** 10.1371/journal.pbio.3000272

**Published:** 2019-06-04

**Authors:** Buzz Baum, Enrico Coen

**Affiliations:** 1 MRC Laboratory of Molecular Cell Biology and the Institute of Physics of Living Systems, University College London, London, United Kingdom; 2 Department of Cell and Developmental Biology, John Innes Centre, Colney Lane, Norwich, United Kingdom

## Abstract

The Internet is rapidly changing the way the results of academic research are communicated within communities and with the wider public. In a push to accelerate change and make the results of research immediately and freely available online for all to read and use, the European Commission, with support from a group of high-profile funders, has proposed a plan to influence the way academic research is published. Here, we discuss the likely impact of this plan on the publishing landscape, the potential benefits, and some possible unintended consequences.

## Abstract 2

To accelerate open access to publicly funded research and to reduce costs of publishing, several funders have proposed to introduce new rules for the way academics publish. Here, two active researchers discuss whether this is a cause for excitement or concern.

## Publish or perish?

Publishing plays a big part in the life of an academic. If someone else publishes similar work first or in a “high impact journal” that reaches a wider audience, it hurts. Such events can determine whether an academic is hired and their research funded. Publishing looms so large because it is the way academics test and share the findings of their research with colleagues and with the wider public. Peer-reviewed papers and their associated data are the main outcome of most funded research. They are a major source of reliable public knowledge that is used to advance technology and to inform future funding and rational policy decisions. This knowledge is a precious commodity in a world that is awash with falsehoods.

## The problems with publishing

A good publication system should meet several criteria: (1) Broad accessibility: Papers, past and present, should be available to be read, and their findings used, by as wide an audience as possible, as soon as possible. (2) High quality peer-review: Papers should be edited and reviewed impartially by relevant experts in the field so as to validate and improve the quality of work published. (3) Clear presentation: Papers should be written and organised clearly. (4) Standards of verification: There should be checks for plagiarism, and obligations to correct the record if errors are revealed (e.g., retractions and/or corrections).

Meeting all of these criteria is not straightforward. As a result, the publishing system that has evolved is complex. The system also suffers from a host of problems, which will be readily apparent to anyone listening to the gossip in the coffee room of a research department. Many of these problems fall hardest on early stage researchers, who have yet to establish their names, and who have more constraints on funding, staff continuity, and publication time than do more established researchers. There are problems with peer review [1]: multiple rounds of revision, biased or ignorant editors and/or reviewers who may be unfairly drawn from sub-sections of the community [2], established academics who by acting as peer reviewers can gain access to the latest research before their more junior peers; problems with plagiarism, fraud, and lack of statistical rigour and reproducibility; problems with presentation: papers that are impenetrable or badly organized; and problems with the way publications are used: journal titles or impact factors being used in isolation as proxies for excellence or lack thereof. There are also problems with the way the system is financed: paywalls, embargoes, high costs of publishing, journals making large profits that are not fed back into the community.

## Evolution or revolution?

Thus, there are many reasons for wanting to see major changes in the way academic publishing works. And changes are afoot. The Internet has brought with it a host of new opportunities and challenges. In return for an open access charge to cover the costs of online publication without subscription, many journals now allow papers to be accessed for free at the time of publication. In addition, most journals allow authors to post a version of their paper online, either prior to peer-review or prepublication, making the work available for all to read [3] [4], leading some to propose a future in which papers are revised and reviewed after being made public [5]. There have been moves toward journals having a more transparent review process [6], in which the reviews, and sometimes names of reviewers, are made public. Journals such as *PLoS ONE* have appeared that have minimal requirements for likely impact in the field, which in any case can only be properly assessed retrospectively. There are efforts by communities to highlight, critique, and review important papers in their field irrespective of where the paper has been posted or published (e.g., F1000 [7] and Pre-lights [8]). Thus, the nature of science communication is changing in dramatic ways.

Given this changing landscape, how can academics, academic institutions, learned societies, funders, and publishers ensure that, as it changes, the system works well to achieve the goals outlined above? This is where Plan S comes in.

Plan S has been drawn up by the European Commission and is being promoted by a host of important research funders from across Europe [9]. The plan focuses on broad accessibility, driven by the view that “free access to all scientific publications from publicly funded research is a moral right of citizens” [10]. It aims to accelerate change in the way the academic publishing market functions by putting rules in place that restrict the type of journals authors can publish in: “After 1 January 2020, scientific publications on the results from research funded by public grants provided by national and European research councils and funding bodies must be published in compliant Open Access journals or on compliant Open Access Platforms” [11].

The idea of making research findings freely available to all immediately on publication is laudable and addresses one of the criteria for a good publication system: broad accessibility. But what might the consequences of Plan S be on academic publishing as a whole?

## Possible consequences of Plan S

If a large number of funders follow Plan S, some currently successful subscription-based journals, including community journals, may find it hard to maintain their income streams, causing them to fold (the precise number depends on the proposed cap that will be implemented on journal Open Access charges) [12–14]. Does the speed of change risk an unnecessary loss of long-standing editorial experience and journal know-how? Plan S avows commitment to quality [10], but how can we ensure that a rapid change doesn’t inadvertently compromise high-quality peer review, rigour, clarity, diversity, and impartiality? Will the notion of elite journals and impact factors disappear? Or, given that academics compete for visibility, prestige, and funding, will these journals simply be replaced with a new elite set of high impact open access journals or platforms? How long will this take, and will the new top journals be any better at selecting the most impactful science than the current ones?

Another possibility is that some funders follow Plan S and others do not, creating a split in the community, with different groups of academics being constrained to submit to different journals [15]. Furthermore, this could lead to a two-tier system in which academics with limited funding are forced to publish in different journals to those with more substantial support (particularly if the cap to open access charges is set high). If some countries sign up for Plan S and others don’t, this could introduce a geographical split in the way science is assessed and published. One of the most treasured aspects of science is its shared standards and unity of purpose. Is this something we wish to put at risk?

## The issue of profits

One of the perceived benefits of Plan S is that the current system leads to subscription journals making excessive profits from science. This seems an inappropriate use of the public and charitable funds that are used to fund science. But is this argument fully justified?

### Non-profit journals

Many journals are run by non-profit organizations. These include the journals run by learned societies or institutions, which recycle surplus into the scientific community by funding meetings, studentships, grants, etc. These journals usually are small scale ventures, rely on subscriptions to remain financially viable, and will likely be hit even harder by Plan S than commercial publishing houses because they lack diversified revenue streams. Plan S may also make it harder for these journals to transition to open access [16].

### Scale of the problem

What is the scale of profiteering and misuse of public funds? Though the global figures for publication costs are large (approximately US$10 billion; [17]), these costs need to be set in relation to the global cost of research and development, which is of the order of 100 times greater (approximately US$1 trillion; [18]). This proportion is also reflected at the institutional level: taking the John Innes Centre as an example, the fraction of the budget spent on journal subscription is approximtely 1%. Although more efforts are required to collect figures on the fraction of science budgets spent on subscriptions for a range of institutions and funding bodies, does an approximately 1% level of spending on subscriptions constitute a misuse of funds when we consider that the communication of science is one of the community’s most important outputs? In response to Plan S, what is to stop established journals compensating for the loss of income in other ways, for example, by raising the costs of accessing historical papers, which have not yet been considered under Plan S?

### Double dipping

Some subscription-based journals use open access charges as an additional source of income. This situation arose, in part, as an unforeseen consequence of funders pushing for full open access publication (Gold). In principle, journals should reduce their subscription charge according to the level of this additional income, but some appear not have not done so [19]. Would stronger regulation (e.g., linking subscription charges to open access income) be a viable and simpler way to prevent abuse of the system, rather than requiring all journals rapidly move to a full open access model as proposed by Plan S?

## The fallacy of taking back control

Many academics would like more control over the publishing system. However, scientists have generally been free to choose where to submit their work. This freedom would be taken away by Plan S.

Many academics also dislike the power that commercial journals wield over their lives. After all, they are forced to fight to publish, pay to publish, review for free, and pay to read their own papers. However, the decisions that commercial journals make are largely based on peer review by academics. So, although it can be comforting to blame the journal, much of the pain of publication comes from the hands of other academics. Of course, editors of journals exert some control by deciding whether to send out for review, choosing referees, and may influence decisions in equivocal cases. But would scientists in the relevant fields make better editors? Full-time researchers have less time to read a wide range of submissions and are influenced by personal knowledge and favouritism just as much (if not more) than professional editors. We currently have a mixed journal economy with some editors being full-time scientists, others full-time editors, some open access, some journal subscription. By reducing diversity would Plan S give scientists more or less control?

Many scientists carry the scars of rejections from prestige journals. The more selective the journal, the more likely that work highly valued by an author has been rejected. It is natural for scientists to blame journals or editors for these rejections. Such personal reactions, although understandable, should not influence policy. Yet it is possible that they have been a factor in the resentment felt towards many journals that gives the notion of taking back control such resonance among some scientists.

## Evolution

The current publication system has evolved over many years. It is far from perfect, but science under this system has flourished. With the Internet, the pace of change has increased dramatically. As a result, open access publishing of peer-reviewed papers (and freely accessible posts) now make up a significant fraction of the sector and are growing [20]. And most academic journals allow authors to self-archive their work on an openly accessible site at some point during the publication process. The system is evolving.

In the face of these developments, although we agree with the aspiration of Plan S to increase access to published work, rushing to force change, starting in approximately half a year from now, without taking into account the potential negative consequences of such change, seems unwise. Plan S threatens to create unnecessary division and uncertainty within a community that, in the current post-truth era, stands out as a haven where shared values and agreed standards of openness and objectivity have enabled steady progress on firm foundations.

We believe it would be prudent to delay forcing through top-down plans (with the threat of sanction for non-compliance) until all the parties involved have been properly consulted, until there is buy-in from other key global players, and until the likely unintended consequences across all aspects of publishing have been fully considered. We welcome that Plan S has invited feedback from the community, but it is unclear which, if any, of its key proposals or timetables are amenable to change. At the same time, the changing landscape of academic publishing should be surveyed, and other measures to widen access explored. These should include finding a way to promote public free access to historic issues of all journals (not just new submissions), looking at mandating preprints as a simple way of removing paywalls for new publications [21] (something that has few cost implications), allowing short embargo periods, and reducing excessive subscription charges.

To conclude, now that Plan S has got everyone’s attention, let’s use the impetus as an opportunity to look afresh at the problems with the current system and get everyone’s input (academics, funders, the public, and publishers) into ways of improving it. All agree broader accessibility is good. The aim should be to achieve it through evolution not revolution.
